# Molecular Regulation of the Melatonin Biosynthesis Pathway in Unipolar and Bipolar Depression

**DOI:** 10.3389/fphar.2021.666541

**Published:** 2021-04-26

**Authors:** Monika Dmitrzak-Weglarz, Ewa Banach, Karolina Bilska, Beata Narozna, Aleksandra Szczepankiewicz, Edyta Reszka, Ewa Jablonska, Paweł Kapelski, Maria Skibinska, Joanna Pawlak

**Affiliations:** ^1^ Department of Psychiatric Genetics, Poznan University of Medical Sciences, Poznan, Poland; ^2^ Laboratory of Neurobiology, Department of Molecular and Cellular Neurobiology, Nencki Institute, Warsaw, Poland; ^3^ Laboratory of Molecular and Cell Biology, Department of Pediatric Pulmonology, Allergy and Clinical Immunology, Poznan University of Medical Sciences, Poznan, Poland; ^4^ Department of Molecular Genetics and Epigenetics, Nofer Institute of Occupational Medicine, Lodz, Poland

**Keywords:** unipolar and bipolar depression, melatonin pathway, candidate gene polymorphism, melatonin metabolite serum level, transcriptomic profiles

## Abstract

Melatonin is a neurohormone that maintains the circadian rhythms of the body. By regulating the secretion of other hormones and neurotransmitters, it acts as a pleiotropic modulator that affects, for example, reproductive, immune, cardiovascular, sleep, and wake systems and mood. Thus, synthetic melatonin has become an essential component in the treatment of depressive disorders. Although we know the pathway of melatonin action in the brain, we lack comprehensive cross-sectional studies on the periphery of depressed patients. This study aimed to comprehensively analyze the differences between healthy control subjects (*n* = 84) and unipolar and bipolar depression patients (*n* = 94), including an analysis of the melatonin pathway at the level of the genes and serum biomarkers. An innovative approach is a pilot study based on gene expression profiling carried out on clinical and cell culture models using agomelatine and melatonin. We confirmed the melatonin biosynthesis pathway's molecular regulation dysfunctions, with a specific pattern for unipolar and bipolar depression, at the AANAT gene, its polymorphisms (rs8150 and rs3760138), and examined the serum biomarkers (serotonin, AANAT, ASMT, and melatonin). The biological pathway analysis uncovered pathways and genes that were uniquely altered after agomelatine treatment in a clinical model and melatonin treatment in a cell culture model. In both models, we confirmed the immunomodulatory effect of melatonin agents in depression.

## Introduction

Depression in its various forms, especially unipolar (UD) and bipolar (BD) depression episodes, is a common disorder in modern societies, one which severely impairs the quality of life and social functioning. The disease can gradually take a chronic form with a high relapse rate, leading to disability (World Health Organization, 2017). Depression can also contribute to suicide, the second most common cause of death among adolescents and young adults (Wasserman, 2016).

Based on the clinical picture alone, it is difficult to differentiate between depression episodes in terms of UD and BD. Therefore, there are no separate diagnostic criteria in the Diagnostic and Statistical Manual of Mental Disorders (DSM). For years, the search for laboratory markers that would facilitate the differentiation of types of depression has been carried out because incorrect diagnosis results in many years of inadequate, ineffective treatment associated with severe impairment of the patient's functioning and health. For example, the use of antidepressants in bipolar depression requires special care because of the risk of inducing mood changes or inducing mania (Baldessarini et al., 2019). Observations and clinical studies confirm the existence of differences between unipolar and bipolar depression on several levels: age of onset, relapse, family burden (Forty et al., 2008), or temperamental traits (Iasevoli et al., 2013; Fornaro et al., 2018). MRI neuroimaging also confirms that there are differences between the types of depression in terms of activation patterns in neural networks, including the amygdala, anterior cingulate gyrus (ACC), prefrontal cortex (PFC), and striatum during emotional, reward, or cognitive functions (Han et al., 2019). However, the cost and availability of the test limit its use in routine differential diagnoses.

Circadian rhythms form an integral part of the depressive symptom complex (Germain and Kupfer, 2008). Beyond disturbed sleep architecture (poor sleep quality, insomnia, or hypersomnia), a lower amplitude in the daily rhythms of locomotor activity, body temperature, and neurohormone secretion, such as norepinephrine, thyroid-stimulating hormone, and melatonin, is often observed in depressive patients (Mendoza, 2019). Based on these foundations, the chronobiological hypothesis of mood disorders was formulated (Malhi and Kuiper, 2013; Zaki et al., 2018), which became the basis for studying the use of exogenous melatonin in the treatment of depression (Boyce and Hopwood, 2013; Alston et al., 2019).

Melatonin (MEL), also known as the dark hormone, regulates the secretion of other hormones and maintains the body's circadian rhythm. Its pleiotropic effects include sleep modulation and antidepressant, anxiolytic, neuroprotective, anti-inflammatory, reproductive, antihypertensive, and antinociceptive traits, and disorders in each area may contribute to the severity of depression (Mahmood, 2019). In humans, the synthesis of MEL occurs primarily in the pineal gland, a small endocrine gland of specialized cells called pinealocytes. Small amounts of this hormone are also produced by the retina and lens of the eye, the lining of the gastrointestinal tract, and blood cells. The precursor of MEL synthesis is tryptophan (an exogenous amino acid), which is transformed into melatonin through an intermediate product, that is, serotonin. Two main rate-limiting enzymes, serotonin-N-acetyltransferase (AANAT) and acetyloserotonin-O-methyltransferase (ASMT), are involved in the process. The synthesis and release of melatonin are subject to fluctuations in the circadian cycle. The release of the hormone is regulated by postsynaptic receptors located in the hypothalamic suprachiasmatic nuclei (SCN), which receive stimuli from the retina and are considered the anatomical center of the biological clock associated with the diurnal light cycle. Melatonin achieves multiple biological effects by activating two membrane G-protein–coupled receptors, MT1 and MT2, and the retinoid orphan nuclear hormone receptor family (RZR/ROR). The primary mechanism of MEL synthesis and secretion in the brain is well understood and has been described in many previous studies (Singh and Jadhav, 2014; Dmitrzak-Weglarz and Reszka, 2017; Tordjman et al., 2017).

In both UD and BD depressive disorders, a decrease in nocturnal melatonin secretion and delayed peak secretion (phase shift) were observed (Khaleghipour et al., 2012; Melo et al., 2017). Treatment with exogenous MEL did not produce the expected antidepressant effects in humans (Carman et al., 1976). On the other hand, elevated levels of MEL in the blood plasma were noted after antidepressant treatment (Palazidou et al., 1992). In turn, a significant improvement in the state of depression and anxiety was observed after the administration of agomelatine (Salva and Hartley, 2012; Konstantakopoulos et al., 2020).

Agomelatine is a melatonergic agonist (MT1 and MT2 receptors) and an antagonist of 5-HT2C and 5-HT2B receptors. Agomelatine restores the circadian rhythm *via* the MT1 and MT2 receptors present in the suprachiasmatic nucleus. In turn, the antidepressant effect of agomelatine is related to its simultaneous influence on melatoninergic receptors and the 5-HT2C receptor. This action leads to an increase in dopaminergic and noradrenergic transmission in the prefrontal cortex, an increase in the 5-HT1A serotonergic receptor's activation without changes in its sensitivity, and normalization of disturbed circadian rhythms and sleep disorders. The antidepressant effect of agomelatine is also attributed to its neuroprotective properties and the reduction of glutamatergic activity resulting from its 5-HT2C receptor antagonism. It has been suggested that by acting on melatoninergic and serotonergic 5-HT2C receptors, agomelatine may also have an antianxiety effect and positively affect sexual behavior. The detailed mechanism of action, pharmacokinetics, and clinical characteristics of agomelatine have been characterized elsewhere (De Berardis et al., 2013; Fornaro et al., 2014; De Berardis et al., 2015).

Many melatonin derivatives are currently being used in the treatment of sleep disorders and depressive symptoms, many of which are available over the counter. However, we still do not fully understand the MEL pathway operation, although the available data have been updated in recent years (Hickie and Rogers, 2011).

Depressive disorders are strongly associated with genetic susceptibility (Serretti, 2017). Most of the associated studies have focused on the CLOCK genes that regulate the feedback loop of the master biological clock (Dmitrzak-Weglarz et al., 2015; Dmitrzak-Weglarz et al., 2016). Studies of single nucleotide polymorphisms (SNPs) in key genes of the melatonin biosynthesis and metabolism pathway may help clarify the pathological mechanisms of melatonin production associated with depressive disorders. However, the associated studies to date have focused on only two genes that encode the enzymes involved in the conversion of serotonin to melatonin: serotonin-N-acetyltransferase, AANAT (rs3760138, rs4238989, and rs8150) and acetyloserotonin-O-methyltransferase, ASMT (rs4446909) (Kripke et al., 2011). The associated studies on melatonin-related genes are insufficient and often not replicable in mental disorders; in depression, in some cases, there is a complete lack of them.

In recent years, expression microarrays have been used intensively as tools to search for biomarkers that change in disease or under the influence of applied treatment. Nonetheless, isolated reports refer to melatonin pathway profiling (Ha et al., 2006). Current modern microarray and bioinformatic methods may allow us to discover entirely new genes and pathways involved in MEL biosynthesis.

Although MEL was discovered more than 60 years ago, its relationship to depression is still not fully understood. Despite the well-established metabolism of MEL in the brain through animal models and postmortem studies, there is no comprehensive study of this pathway and its putative alterations in the peripheral blood in humans suffering depression.

Given the above, the purpose of this study is to investigate whether

- SNPs of selected candidate genes related to MEL biosynthesis pathway biomarkers (MBPBs) are associated with the risk of UD and BD depression
- different patterns of blood serum levels of key MBPBs feature in an episode of depression, depending on the type of depression, and after the treatment
- there are relationships between the studied polymorphisms and the levels of MBPBs in the blood serum
- screening with the use of expression microarrays allows determination of differentially expressed genes (DEGs) between agomelatine-treated vs. nontreated depressed patients (clinical model) and between MEL-treated vs. nontreated hippocampal neurons (cell culture model). We used primary cultures to analyze the specific cellular pathways after MEL treatment in a controlled environment. In this case, hippocampal neurons are used to study specific neuronal pathways that would otherwise prove difficult, if not impossible, to analyze in the intact brain (Seibenhener and Wooten, 2012).

This study is a pioneering study in the comprehensive analysis of peripheral blood MEL metabolism. In this project, we propose a comprehensive study of the MEL pathway at the levels of the genes, transcripts, and proteins through all MEL synthesis and metabolism stages.

## Material and Methods

The study consisted of the following parts: genetic association analysis from peripheral blood, measurement of MEL biosynthesis pathway biomarkers (MBPBs) in serum, expression profiling analysis in PBMC after agomelatine treatment taken from depressed patients (clinical model), and after MEL treatment of mice primary hippocampal neuron culture (cell culture model).

### Participants

All the participants were of Caucasian origin, and they were native Polish population. The study followed the rules of the Declaration of Helsinki and complied with Good Clinical Practice guidelines. The Bioethics Committee at the Poznan University of Medical Sciences (PUMS) (resolution no. 758/17) approved the study protocol. All participants gave written informed consent to participate in the study.

Recruitment was conducted in 2017–2019 at the Department of Adult Psychiatry, PUMS. Participants were recruited from among patients hospitalized for an episode of depression in the course of F.31 (bipolar disorder) or F.33 (recurrent depressive disorders) according to ICD-10 classification. A consensus lifetime diagnosis was made by two psychiatrists according to the ICD-10 and DSM-IV criteria, using SCID (Structured Clinical Interview for DSM Disorders) (First et al., 1996) and OPCRIT (the Operational Criteria Diagnostic Checklist) (McGuffin et al., 1991). The Hamilton Depression Rating Scale (HDRS17) was used to assess the severity of depression symptoms. A score >20 was required for inclusion in the study, indicating at least moderate severity of depression (pretreatment, Pre-T). A scoring <8 was required for achieving clinical remission or at least 50% reduction at HDRS, defined as treatment response (posttreatment, Post-T) (Hamilton, 1960). All patients after inpatient admission were treated with antidepressants and/or mood stabilizers according to doctors’ choice (Rybakowski, 2018). Patients enrolled in the study also had to meet the following inclusion/exclusion criteria (World Health Organization, 2017): age 18–65 years (Wasserman, 2016), at least the second episode in lifetime history (Baldessarini et al., 2019), and no chronic or acute somatic or neurological diseases. We offered participation in the study to a total of 110 patients. The diagnosis was not confirmed in 4 subjects, 3 subjects met the exclusion criteria, 2 subjects withdrew their consent to participate in the study, 4 subjects did not achieve minimal clinical improvement, and 3 subjects failed to obtain biological material for laboratory analyses.

The age-matched healthy control group was recruited from healthy volunteers recruited at the Nofer Institute of Occupational Medicine in Lodz. The control group was psychiatrically screened using a short diagnostic structured interview—the Polish version of the Mini International Neuropsychiatric Interview scale—to exclude those with any serious mental health problems (Lecrubier et al., 1997). Controls enrolled in the study also had to meet the following inclusion/exclusion criteria (World Health Organization, 2017): age 18–65 years (Wasserman, 2016), no family history of psychiatric disorders in first-degree relatives (Baldessarini et al., 2019), and no chronic or acute somatic or neurological diseases.

Eventually, the study was performed on 94 patients (UD, *n* = 41 and BD, *n* = 53) and 84 healthy controls (HC, *n* = 84) of both sexes.

### Biological Material Collection

Blood samples from all subjects were restricted to those collected between 7.00 and 9.00 a.m. (after overnight fasting) to carry out all molecular analysis using the same procedures and under the same conditions to avoid daytime fluctuation and any pre-laboratory errors that could influence the final results.

### Genotyping

The DNA was extracted from 5 ml of EDTA anticoagulated whole blood using the salting-out method (Miller et al., 1988). The SNP selection was based on the criteria described previously (Dmitrzak-Weglarz and Reszka, 2017). The 15 polymorphisms in the *AANAT*, *ASMT*, *MTNR1B*, *MTNR1A*, *CYP1A2*, and *CYP1B1* genes have been identified with the use of KASP™ genotyping assays provided by LGC Genomic Laboratory (Hoddesdon, United Kingdom). In summary, KASP genotyping assays are based on competitive allele-specific PCR. Allelic discrimination is based on the competitive binding of two allele-specific primers labeled with FAM™ or HEX™ dye. The detailed described protocols can be found in the Biosearchtech Producers User Guide.^1^ ^,^ ^2^ ^,^ ^3^ Information about the analyzed SNPs and primers is shown in Table 1.

**TABLE 1:** Information about the studied polymorphisms.

Gene name ID gene	rs#	Alternative	Ancestral/reference allele	MAF^b^	Primer_AlleleX	Primer_AlleleY
*AANAT*–(serotonin-N-acetyltransferase) ID:15	rs8150	G > C, A^a^	G	C = 0.3201	AAT​ATT​ATT​TAA​TCC​ATG​AGA​CAT​CGT​CCA	ATT​TAA​TCC​ATG​AGA​CAT​CGT​CCG
	rs3760138	G > T, A^a^	G	G = 0.4781	GGG​GTG​CAG​GAT​GGG​GTG​TA	GGG​TGC​AGG​ATG​GGG​TGT​G
	rs4238989	C > G	C	C = 0.4423	CCC​TCG​GAG​TCT​TGG​ACA​AG	CCC​TCG​GAG​TCT​TGG​ACA​AC
*ASMT*–acetyloserotonin-O-methyltransferase ID:438	rs4446909	G > A	G	A = 0.286	GAA​CTG​ATC​CCA​AAC​CAA​TAT​GCT​C	AGA​ACT​GAT​CCC​AAA​CCA​ATA​TGC​TT
	rs5989681	G > C, A^a^	G	C = 0.302	AGG​GGA​TCA​CAA​AGC​GTG​TGG​T	GGG​ATC​ACA​AAG​CGT​GTG​GC
*MTNR1B*–melatonin receptor 1B ID:4544	rs4753426	C > T, A^a^	T	T = 0.4980	AAT​ACA​ACA​TAT​TTG​TGA​TTA​ATC​CAT​GCC	CAA​TAC​AAC​ATA​TTT​GTG​ATT​AAT​CCA​TGC​T
	rs10830963	C > G,T^a^	C	G = 0.2883	GGC​AGT​TAC​TGG​TTC​TGG​ATA​GG	GGC​AGT​TAC​TGG​TTC​TGG​ATA​GC
	rs156244^c^	C > A,G^a^,T^a^	C	C = 0.0010	ATT​AAC​GTC​ATC​ATT​AAA​ATA​ATA​AAA​CCC​AAA	AAC​GTC​ATC​ATT​AAA​ATA​ATA​AAA​CCC​AAC
	rs6483221	C > T,A^a^,G^a^	C	C = 0.1968	AAA​AGA​TCA​GAT​ATG​TTA​ACA​TTC​TTG​ATA​AG	CAA​AAG​ATC​AGA​TAT​GTT​AAC​ATT​CTT​GAT​AAA
*MTNR1A*- melatonin receptor 1A ID:4543	rs2119882	C > T		T = 0.3837	CCC​CAA​TCC​CAT​TTC​GCA​TTT​GG	CCC​CAA​TCC​CAT​TTC​GCA​TTT​GA
	rs12506228	C > A	C	A = 0.2913	GAA​ACT​AAG​ATG​GGT​AGA​AAG​TCA​GAT	AAA​CTA​AGA​TGG​GTA​GAA​AGT​CAG​AG
*CYP1A2*–cytochrome P450 1A2 ID:1544	rs2470890	T > C	T	C = 0.35424	CAG​AAT​GGT​GGT​GTC​TTC​TTC​AA	CAG​AAT​GGT​GGT​GTC​TTC​TTC​AG
	rs762551	C > A, G^a^	C	C = 0.35424	CCA​TCT​ACC​ATG​CGT​CCT​GT	CCA​TCT​ACC​ATG​CGT​CCT​GG
	rs2472304	A > G	G	G = 0.4006	GTA​GAC​TGA​ACA​AAC​AAC​CTG​GGT​T	AGA​CTG​AAC​AAA​CAA​CCT​GGG​TC
*CYP1B1*–cytochrome P450 1B1 ID:1545	rs1056836	G > C, (exon) Leu432Val	G	C = 0.4434	GTG​GTC​TGT​GAA​TCA​TGA​CCC​AC	GTG​GTC​TGT​GAA​TCA​TGA​CCC​AG

a Allele not identified in studied population.

b 1000GENOMES Europe population based on GRCh38. p12 version.

c Variant not polymorphic in studied population.

### Serum Samples

5 ml of venous blood was withdrawn into anticoagulant-free tubes. After 30 min of incubation, serum was separated by centrifugation, aliquoted, and after two-stage freezing, at −20 and then −80°C, stored until further analyses. For measurement of the MBPB enzyme-linked immunosorbent assays were performed using the commercially available tests. The detailed description is presented in Supplementary Excel File_1–ELISA Tests.

All ELISA tests were performed according to the manufacturer's instructions, without any modifications. The status of all used tests was for research use only (RUO). All samples and standards were run in duplicates, and the mean value of the two assays was used for statistical evaluation. Optical density was read with a spectrophotometric plate reader (Asys UVM 340 Microplate Reader from Biochrom Ltd., Cambridge, United Kingdom) for a wavelength of 450 nm ± 10 nm. A four-parameter logistic ELISA curve fitting was used to assay concentration in the tested samples.

### RNA Sample Processing

#### Clinical Sample

To perform pilot profile gene expression on microarrays between agomelatine-treated and non–agomelatine-treated patients, we selected only women who did not smoke, did not take hormonal drugs, and did not work in shifts. The detailed description is presented in Supplementary Excel File_1–Microarray Clinical Samples.

4 ml of venous blood was withdrawn into an EDTA anticoagulated tube and immediately drawn through a LeukoLOCK™ filter, washed with 1xPBS (Life Technologies, United States), and saturated with RNAlater^®^ (Life Technologies, United States). Then, the inlet ports of the filter were sealed, and the filter was stored at −80°C until processing (Gautam et al., 2019).

RNA isolation and purification were performed according to alternative protocol for extraction of RNA from PBMC captured on LeukoLOCK™ filters.^4^

#### Cell Culture

Primary cultures of dissociated hippocampal neurons (HNs) were prepared from FVB mice at postnatal day 0 (P_0_), according to the standard protocol (Seibenhener and Wooten, 2012). The neurons were plated at a density of 1.6 × 10^5^ cells per well in 12-well plates. Cell cultures were allowed to settle at 37°C in a 5% CO_2_ atmosphere. Cultured neurons from two independent cultures were stimulated for 24h with 10 µM MEL dissolved in ethanol at DIV19. The nontoxic concentration of MEL was defined based on previously published data (Park et al., 2007). Total RNA was extracted from cell culture samples using the RNeasy^®^ Mini Kit (QIAGEN). Cell line testing is not an experimental procedure. The animals used to obtain the primary lines were treated under Directive 2010/63/EU (Appendix no. VI) by qualified staff.

### Microarray-Based Gene Expression Profiling

RNA concentration and purity were evaluated using a NanoDrop 2000c UV-Vis spectrophotometer (Thermo Scientific, United States). The RNA integrity number (RIN) was measured for each sample using Agilent 2100 Expert software. Samples with a RIN value of 6 and higher were further processed (Schroeder et al., 2006). The SurePrint G3 Human Gene Expression v3 8 × 60K Microarray (Agilent, United States) was utilized in this study. The arrays were processed according to the One-Color Microarray Based Gene Expression Analysis protocol v. 6.9.1. The slides were scanned using a SureScan Dx Microarray Scanner (Agilent, United States). The images obtained after scanning were analyzed using Agilent Feature Extraction software v. 12.0.3.1. The analysis included quality control metrics (filtering of outlier spots, background subtraction from features, and dye normalization) and report. An image and a detailed description of the quality control metrics are in Supplementary Excel File_1-QC Metrics.

**FIGURE 1:**
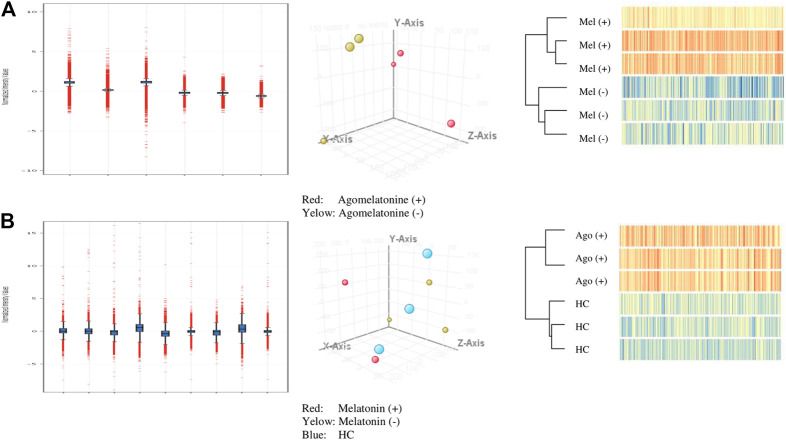
Quality control for analyzed A-cell culture and B-clinical model (normalized intensity value; PCA filtered on flags [detected, not detected]; hierarchical clustering).

The principal component analysis (PCA) was conducted on microarray data (submitted to GEO NCBI resources) using filtered flags [detected, not detected] (Figure 1).

### Statistical and Bioinformatics Analysis

Results were expressed as numbers and percentages, mean ± standard deviation, and median, as appropriate. Statistical analyses were conducted in Statistica v13.3 (StatSoft, Poland), VassarStats (http://vassarstats.net/), and G*Power 3.1 (University Dusseldorf) software. Normality of distribution was assessed with the Shapiro–Wilk test, and equality of variances was checked using Levene's test. Due to the lack of normality for most of the variables, more restrictive nonparametric tests were used. Comparison of two unpaired groups was performed using the U Mann–Whitney test, while comparison of paired groups was performed using the Wilcoxon pair test. Multiple linear regression analysis was used to assess the influence of age and sex on other analyzed clinical and molecular variables. Spearman's rank correlation was used to detect the relationship between variables. Genotype and allele frequencies were compared using the χ^2^-test and Fisher exact test, respectively. All analyzed polymorphisms were at Hardy–Weinberg equilibrium. All tests were two-tailed, and *α* was set at 0.05 (as we compared two groups simultaneously). To determine *a priori* minimal sample size, we used G*Power 3 software (Faul et al., 2007; Faul et al., 2009). Given *α* = 0.05, power (1−*β*) = 0.8, a medium effect size, and the total desired sample size was as follows:

− *n* = 108 for genetic analyses and the χ^2^-test (w = 0.3),
− *n* = 134 for the Mann–Whitney test (*d* = 0.5),
− *n* = 35 for the Wilcoxon pair test (dz = 0.5),
− *n* = 68 for multiple linear regression analysis (*f* ^2^ = 0.15).

Therefore, the study included adequate sample size, and the statistical power was appropriate to detect significant differences in the studied groups.

### Differentially Expressed Genes

The data obtained after extraction were further analyzed using GeneSpring 14.9.1 (Agilent, United States). The analysis aimed to determine differentially expressed genes (DEGs) in the PBMC of the patients treated with agomelatine and primary HN culture treated with MEL.

To identify up- and downregulated gene lists, we used restricted cutoff with fold change >2 and a significant *p*-value of <0.05 according to previous indications (Dalman et al., 2012). Significant differences in gene expression were determined *via* moderated Student's t-test. The differences were considered statistically significant at *p* < 0.05. According to the Benjamini–Hochberg procedure, the obtained *p*-values were corrected using the false discovery rate (FDR) as multiple testing correction methods (Cheng and Pounds, 2007).

### Pathway Analysis

Lists of differentially expressed genes, with the restricted cutoff described above, were uploaded to the DAVID 6.8—Available Online Database for Annotation, Visualization, and Integrated Discovery Classification System (DAVID; https://david.ncifcrf.gov/home.jsp) to analyze their functional affiliations and participation in curated pathways (Huang et al., 2009). We used the Expression Analysis Systematic Explorer (EASE) tool to automate biological theme determination for a downloaded list of genes. We used the analysis inclusion threshold as the EASE Score—a modified Fisher exact *p* < 0.05 (Kleber et al., 2015). Ontologies and pathways were assigned independently to upregulated and downregulated gene lists. Annotations were limited to HGNC gene symbols and the *Homo sapiens* genome as a background (Wain et al., 2002). The Kyoto Encyclopedia of Genes and Genomes (KEGG) was used as a reference database (Kanehisa et al., 2019). To present significant protein–protein association networks, we used STRING v11 software (Szklarczyk et al., 2019).

## Results

### Sample Description

The clinical characteristics of patients and control subjects are presented in Table 2.

**TABLE 2:** Clinical description of the study population.

	BD (*n* = 53)			UD (*n* = 41)			HC (*n* = 84)			M–W test		
	Mean	SD	Median	Mean	SD	Median	Mean	SD	Median	BD vs. UD	BD vs. HC	UD vs. HC
Age	41.91	13.87	43.00	43.49	15.99	47.00	43.11	10.91	40.00	0.616	0.845	0.716
Years of education	13.22	2.87	12.00	13.72	2.83	12.00	15.86	2.06	17.00	0.488	**1.047E−06**	**1.712E−04**
BDI	34.47	9.44	35.50	30.23	10.83	29.00	4.04	2.95	4.00	**0.037**	**1.936E−29**	**6.280E−27**
BMI	24.46	4.88	23.10	24.89	4.96	24.16	23.80	3.20	23.62	0.679	0.764	0.428
Onset of illness	27.57	9.80	26.00	35.16	14.49	33.00				**0.013**		
Number of hospitalizations	7.35	15.76	3.00	1.89	2.16	1.00				**2.470E−04**		
Duration of current hospitalization (days)	50.64	24.92	50.00	43.32	16.02	43.00				0.283		
HDRS Pre-T	25.93	6.60	24.00	25.50	4.76	25.00				0.783		
HDRS Post-T	3.17	2.50	3.00	4.41	4.19	3.00				0.316		
W-P test (HDRS Pre-T vs. Post-T)			**7.623E−09**			**5.392E−07**						

BD, bipolar disorder; UD, unipolar disorder; HC, healthy control; n, number; SD, standard deviation; M–W test, Mann–Whitney *U* test; W-P test, Wilcoxon pair test.

Significant p-values are indicated in bold (significance considered p < 0.05).

In our group, both the BD and UD patients had significantly shorter education (*p* < 0.001) and higher BDI scores (*p* < 0.001) than the HC subjects. Bipolar patients demonstrated a higher score of BDI (*p* = 0.037), an earlier age of onset (*p* = 0.013), and more hospitalizations (*p* < 0.001) than UD patients. All patients achieved a significant and required posttreatment improvement in depression symptoms on the HDRS scale (*p* < 0.001 for BD and UD, respectively). The duration of current hospitalizations did not differ significantly between bipolar and unipolar depression (*p* = 0.283).

During recruitment, male patients more often refused to take part in the study, resulting in the sex ratio disproportion: BD: *n* = 7 (13.2%), UD: *n* = 4 (9.8%), HC: *n* = 13 (15.5%). To control sex and age as confounding factors, we performed multiple linear regression analysis. This analysis showed no significant effects of age (*p* = 0.821) or sex (*p* = 0.472) on clinical and molecular variables (F (2,178) = 0.293; *p* = 0.746; *R* ^2^ = 0.003). Thus, in further analyses, groups were not subdivided according to age and sex of participants.

Out of 15 analyzed polymorphisms related to the MEL pathway, only rs156244 in the *MTNR1B* gene turned out to be non-polymorphic in the analyzed population. No deviation from the Hardy–Weinberg equilibrium law was observed for the rest of the analyzed polymorphisms. Case–control comparisons between patients with bipolar depression and control subjects showed an association of two AANAT polymorphisms (rs8150; *p* = 0.028 and rs3760138; *p* = 0.043). For the other analyzed polymorphisms, we did not find any significant differences in genotype and allele frequencies between the compared groups of patients and controls (Supplementary Excel File_1-Genotypes & Alleles).

### Serum Expression of MEL Biosynthesis Pathway Biomarkers (MBPBs)

**FIGURE 2:**
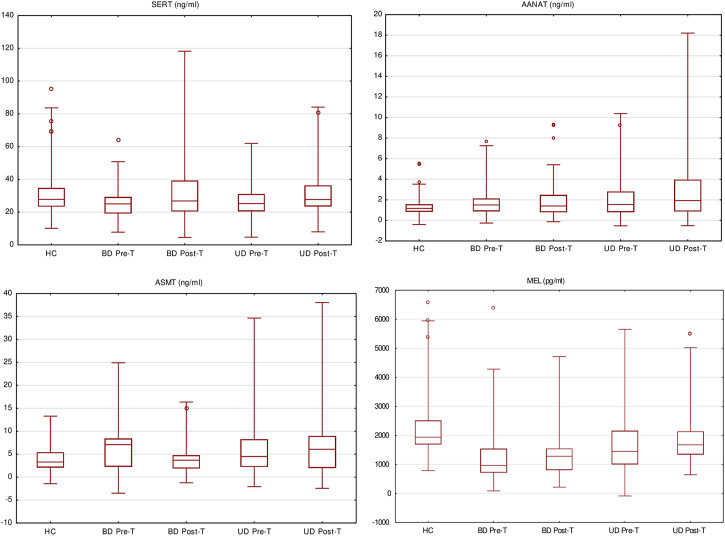
Melatonin biosynthesis pathway biomarkers’ serum level pre- and posttreatment (box and whisker plots showing median values, interquartile ranges (box), non-outlying values (whiskers), and outlier points (dot)).

The serum concentrations of MBPBs are presented in Table 3 and Figure 2.

**TABLE 3:** Comparisons of melatonin biosynthesis pathway biomarkers in the serum level.

		BD				UD				HC				Comparisons				
		SERT ng/ml	AANAT ng/ml	ASMT ng/ml	MEL pg/ml	SERT ng/ml	AANAT ng/ml	ASMT ng/ml	MEL pg/ml	SERT ng/ml	AANAT ng/ml	ASMT ng/ml	MEL pg/ml	M–W test *p*-value	SERT ng/ml	AANAT ng/ml	ASMT ng/ml	MEL pg/ml
Pre-T	Mean	26.03	2.07	5.97	1267.85	31.28	2.29	5.19	1508.37	36.68	1.60	4.01	2329.27	BD vs. HC	**0.0101 (↓)**	**0.0334 (↑)**	**0.0142 (↑)**	**<0.0001 (↓)**
	SD	9.74	2.07	4.11	1018.38	16.30	2.45	6.31	1266.95	17.22	2.56	2.44	1200.01	UP vs HC	0.4275	0.1479	0.9265	**<0.0001 (↓)**
	Median	25.02	1.50	7.09	965.70	26.81	1.40	3.69	1282.52	17.82	1.17	3.30	1938.24	BD vs. UD	0.2259	0.8534	0.1421	0.2364
Post-T	Mean	27.39	2.23	7.70	1873.95	31.01	2.70	6.82	2265.82					BD vs. HC	0.0965	0.0643	0.0955	**0.0001 (↓)**
	SD	8.43	1.99	10.69	1928.45	12.79	2.65	6.13	1684.21					UP vs HC	0.8961	**0.0079 (↑)**	**0.0194 (↑)**	**0.0159 (↓)**
	Median	25.30	1.42	4.57	1519.71	27.68	1.93	6.08	1674.58					BD vs. UD	0.1759	0.4982	0.5802	0.1160
W-P test *p*-value		**0.0173 (↑)**	0.1992	0.5313	**0.0018 (↑)**	0.2137	0.2845	0.9250	**0.0006 (↑)**									

UD, unipolar disorder; BD, bipolar disorder; Mann–Whitney *U* test; Wilcoxon pair test; (↑)—the arrow shows the direction of changes in the concentration of the tested metabolite in relation to the reference group.

Significant p-values are indicated in bold (significance considered p < 0.05).

**TABLE 4:** Differentially expressed genes (DEGs) in studied models.

Model	Total DEGs, *p* < 0.05, FC > 2	Up	Down	DAVID/KEGG^a^	Up	Down
Clinical model						
Agomelatine (+) vs. HC	4648	2217	2431	1275	542	733
Agomelatine (−) vs. HC	3999	567	3432	803	152	651
Common and different genes (Venn diagram)	3224	1688	1536	947	402	545
Cell culture model						
Melatonin (+) vs. melatonin (−)	18,489	762	17727	13,105	762	5343

FC, fold change; PBMC, peripheral blood mononuclear cells; HN, hippocampal neurons.

a Functionally known by the DAVID/KEGG database.

We observed significant differences in the concentration of all the measured MBPBs between pretreatment BD and HC. In BD patients, serotonin and MEL concentration were significantly decreased (SERT *p* = 0.0101; MEL *p* < 0.0001), while both measured enzymes were increased (AANAT *p* = 0.0334; ASMT *p* = 0.0142). After treatment (comparison between pre- and posttreatment), we observed an increase in all biomarkers’ concentration, although it was significant only for serotonin (SERT *p* = 0.0173) and MEL (MEL *p* = 0.0018). A recomparison of posttreatment BD vs. HC has revealed the existence of a still significantly lower level of MEL concentration (MEL *p* = 0.0001).

Comparison between pretreatment UD and HC showed that similarly to BD, the concentrations of serotonin and MEL were decreased, and the enzymes increased, although the significant difference was only in the concentration of MEL (MEL *p* < 0.0001). After treatment (pre- vs. posttreatment), we observed a significant increase in MEL concentration (MEL *p* = 0.0006). Recomparison of posttreatment UD vs. HC revealed that the differences in the concentrations of both enzymes worsened in UP patients (AANAT *p* = 0.0079; ASMT *p* = 0.0194). Also, a significantly lower level of MEL concentration (MEL *p* = 0.0159) was still observed.

### Serum Concentration of MBPBs Dependent on Genotype Carriers

In the patients, with regard to the type of depression and the disease's state, we found several significant dependencies: rs2119882 and ASMT (*p* = 0.0012), rs3760138 and MEL (*p* = 0.0330), rs762551 and AANAT (*p* = 0.0022), rs3760138 and AANAT (*p* = 0.0094), rs12506228 and AANAT (*p* = 0.0269), and rs1056836 and ASMT (*p* = 0.0267) (Supplementary Excel File_1-Spearman's Rank Correlation).

**FIGURE 3:**
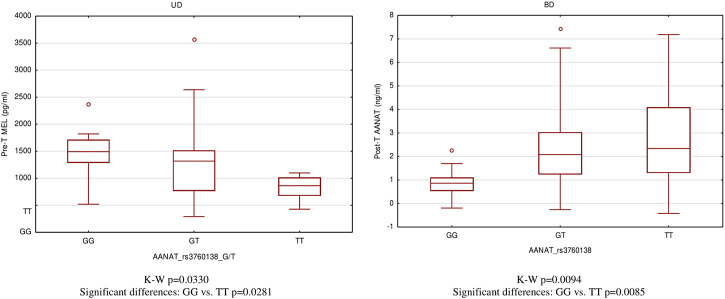
Differences of MEL and AANAT serum level between *AANAT* rs3760138 genotypes.

For significantly correlated pairs, we provided comparisons of serum concentration of MBPBs between genotypes. Only the rs3760138 polymorphism of the AANAT gene, whose GG genotype, is associated with the highest concentrations of MEL in the pretreatment state (*p* = 0.0281), while the TT genotype is associated with the highest concentrations of AANAT in the posttreatment state (*p* = 0.0085), turned out to differentiate the type of depression and the disease state (Figure 3).

### Differentially Expressed Genes (DEGs)

#### Clinical Model

**FIGURE 4:**
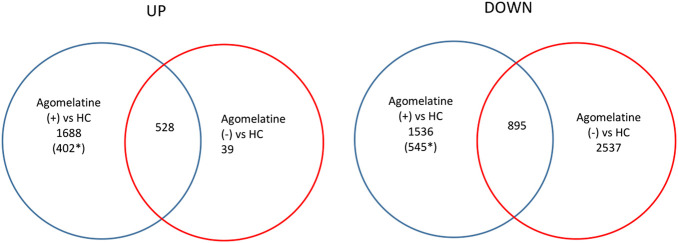
Venn diagrams representing unique and shared differential gene expression profiles of agomelatine (+) & (−) patients, numbers of genes upregulated (UP) and downregulated (DOWN), blue ring—number of genes uniquely up- or downregulated in agomelatine (+), and red ring—number of genes uniquely up- or downregulated in agomelatine (−). Overlapping parts of the diagram—the number of shared genes uniquely up- or downregulated between groups of patients. (For interpretation of the references to color in this figure legend, the reader is referred to the Web version of this article.) *functionally known by the DAVID/KEGG database.

We found numerous differentially expressed genes (DEGs) in the clinical model. For example, in agomelatine (+), we discovered 2217 upregulated and 2431 downregulated genes with minimal FC > 2. After gene ontology term enrichment, only 542 upregulated and 733 downregulated genes were functionally known by the DAVID/KEGG database. We used the lists of the upregulated and downregulated genes in agomelatine (+) and (-) as a set of genetic markers. The comparison of these collections allowed for identifying unique and shared gene expression profiles between the compared groups of patients. For comparisons, Venn diagrams were constructed (Figure 4). The list of unique and shared genes was processed again in the DAVID 6.8 database to obtain renewed data.

#### Cell Culture Model

A fold change of more than 2 was observed for 762 upregulated and 17729 downregulated genes in MEL (+) vs. MEL (-) treated cell cultures. The 762 up- and 5343 downregulated genes were functionally known by the DAVID/KEGG database.

#### Gene Ontology Term Enrichment

In Table 5, we present a summary of gene set enrichment and pathway analysis.

**TABLE 5:** Gene set enrichment.

	Up				Down			
	Gene ontologies			KEGG_P	Gene ontologies			KEGG_P
	BP	CC	MF		BP	CC	MF	
Agomelatine (+) vs. HC	67	10	43	6	667	65	45	37
Agomelatine (−) vs. HC	39	14	16	1	305	41	70	20
Unique for agomelatine	76	6	30	3	639	52	30	45
Melatonin (+) vs. (−)	9	4	2	—	213	28	63	10

BP, biological process; CC, cellular component; MF, molecular function; KEGG_P, KEGG pathway; analysis inclusion threshold, EASE score, a modified Fisher exact *p* < 0.05.

Genes upregulated in agomelatine (+) significantly enriched 120 Gene Ontologies: 67 from the Biological Process (BP) category, 10 from the Cellular Component (CC) category, and 43 from the Molecular Function (MF) category. Upregulated genes significantly enriched 6 KEGG pathways.

Genes downregulated in agomelatine (+) enriched 777 Gene Ontologies: 667 from the Biological Process, 65 from the Cellular Component, and 45 from the Molecular Function categories. Downregulated genes significantly enriched 37 KEGG pathways.

The factsheet for Gene Ontology (GO) term enrichment and pathway analysis provided in all individual analyzed types of models is presented in Table 5.

The complete list of significantly altered GO terms and pathways was included in Supplementary Excel File_2 with the appropriate Excel spreadsheet.

#### Pathway Analysis and Gene Interaction Network

**FIGURE 5:**
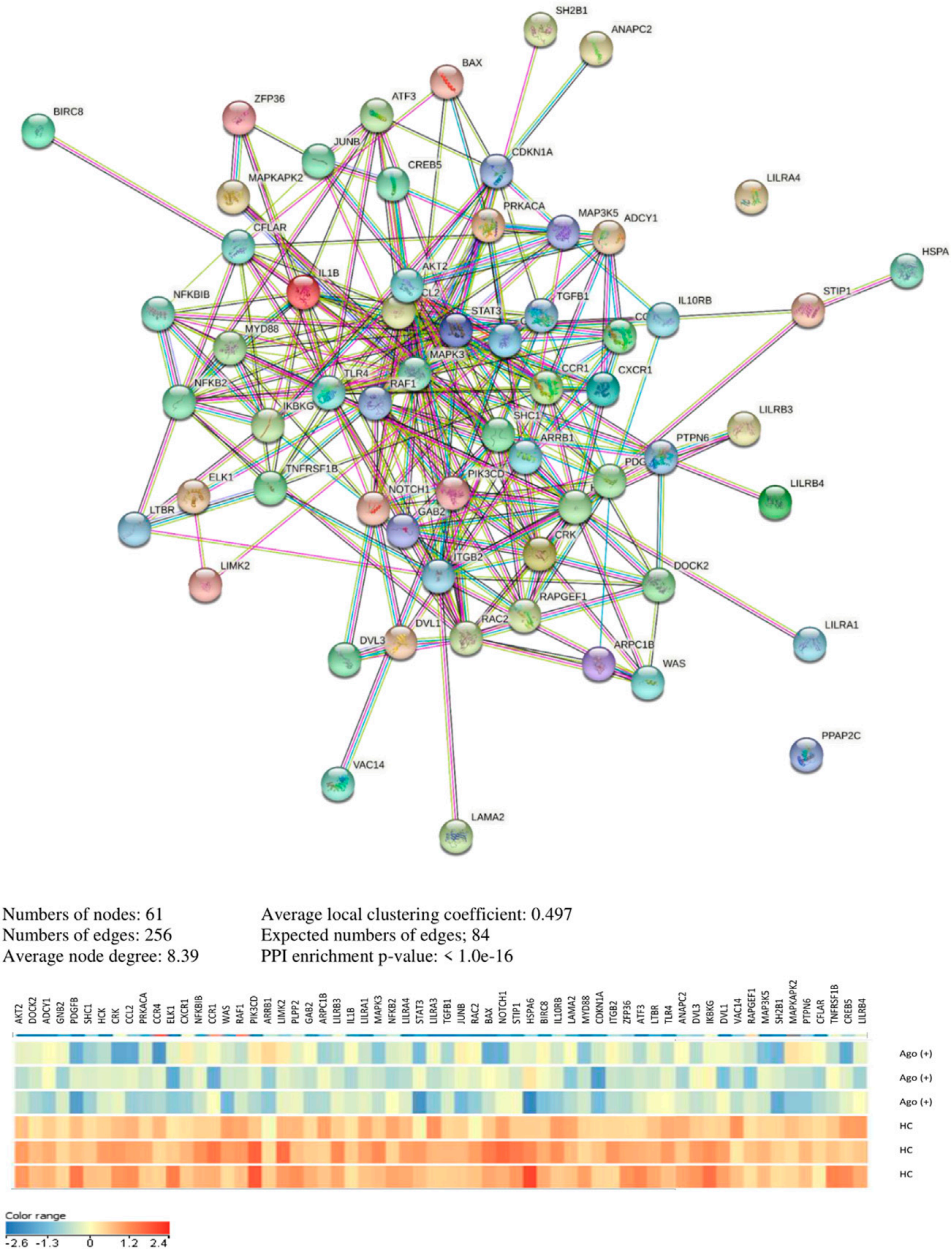
Interaction network of unique genes for agomelatine treatment.

In Supplementary Excel File_1-KEGG Pathways & Genes, we present a significant pathway and genes in both analyzed clinical and cell culture models. The pathway and genes that pass FDR correction are presented in Table 6. Interaction analyses of genes creating unique pathways for agomelatine treatment showed 61 nodes, 256 edges, 8.39 average node degree, and 0.497 average local clustering coefficient (PPI enrichment *p*-value< 1.0e−16) (Figure 5). Approximately 60 proteins that had interacted together were found.

**TABLE 6:** KEGG pathways pass after FDR correction.

	KEGG pathway	Genes in pathway	*p*-value	FDR
	Agomelatine (+) vs. HC	Downregulated genes		
	hsa04666: Fc gamma R-mediated phagocytosis	*AKT2, LIMK2, DOCK2, WASF2, HCK, CRK, MAPK3, RAC2, PLPP2, GAB2, ARPC1B, LIMK2, WAS, RAF1, PIK3CD*	0.0001	0.0161
	hsa04062: chemokine signaling pathway	*AKT2, DOCK2, ADCY1, GNB2, IKBKG, SHC1, HCK, CRK, MAPK3, CCL2, PRKACA, CCR4, RAC2, CXCR1, NFKBIB, CCR1, WAS, RAF1, PIK3CD, ARRB1, STAT3*	0.0002	0.0161
	hsa04380: osteoclast differentiation	*AKT2, LILRB3, TYK2, IKBKG, IL1B, LILRA1, MAPK3, NFKB2, LILRA4, FOS, GAB2, LILRB4, LILRA3, PIK3CD, TGFB1, JUNB, LILRA2*	0.0002	0.0161
	hsa04722: neurotrophin signaling pathway	*AKT2, NTRK1, MAP3K5, SHC1, CRK, MAPK3, NTRK2, MAPKAPK2, BAX, RAPGEF1, NFKBIB, RAF1, PIK3CD, NGFR, SH2B1*	0.0008	0.0478
	Agomelatine (-) vs. HC	Downregulated genes		
	hsa04080: neuroactive ligand–receptor interaction	*AVPR2, GABRQ, GRIN2C, GLP2R, TACR1, GPR50, MC5R, PRSS3, VIPR2, LPAR2, GRIK5, NPY5R, CHRNB4, CRHR2, RXFP2, ADORA1, SCTR, GRIA1, MC3R, RXFP3, TRHR, APLNR, PTGER1, GABRE, GABRR3, GHRHR, VIPR2, GRIN2C, NMUR2, GLRA1, GRIN3A*	6.8960E−08	1.5378E−05
	hsa04972: pancreatic secretion	*ADCY6, PLA2G5, CELA2A, PLA2G12B, GNAS, ATP1B2, PLA2G2A, ATP2B3, ATP2A1, SCTR, ATP2B2, PRSS3*	0.0002	0.0221
	Unique pathways for agomelatine treatment	Downregulated genes		
	hsa04062: chemokine signaling pathway	*AKT2, DOCK2, ADCY1, GNB2, IKBKG, SHC1, HCK, CRK, MAPK3, CCL2, PRKACA, CCR4, RAC2, CXCR1, NFKBIB, CCR1, WAS, RAF1, PIK3CD, ARRB1, STAT3*	3.3486E−06	0.0007
	hsa04666: Fc gamma R-mediated phagocytosis	*AKT2, LIMK2, DOCK2, HCK, CRK, MAPK3, RAC2, PLPP2, GAB2, ARPC1B, LIMK2, WAS, RAF1, PIK3CD*	1.9636E−05	0.0020
	hsa04380: osteoclast differentiation	*AKT2, LILRB3, IKBKG, IL1B, LILRA1, MAPK3, NFKB2, LILRA4, GAB2, LILRB4, LILRA3, PIK3CD, TGFB1, JUNB*	0.0004	0.0277
	hsa05220: chronic myeloid leukemia	*AKT2, CDKN1A, GAB2, RAF1, PIK3CD, IKBKG, SHC1, CRK, MAPK3, TGFB1*	0.0006	0.0309
	hsa05020: prion diseases	*ELK1, BAX, NOTCH1, IL1B, MAPK3, STIP1, PRKACA*	0.0008	0.0327
	hsa05145: toxoplasmosis	*AKT2, HSPA6, BIRC8, IKBKG, IL10RB, LAMA2, MAPK3, MYD88, NFKBIB, TLR4, STAT3, TGFB1*	0.0011	0.0361
	hsa05166: HTLV-I infection	*AKT2, ITGB2, ELK1, ADCY1, ZFP36, IKBKG, ATF3, LTBR, NFKB2, PRKACA, CDKN1A, BAX, ANAPC2, PIK3CD, DVL3, PDGFB, TGFB1, DVL1, VAC14*	0.0020	0.0488
	hsa04722: neurotrophin signaling pathway	*AKT2, BAX, NFKBIB, RAPGEF1, MAP3K5, RAF1, PIK3CD, SHC1, CRK, MAPK3, SH2B1, MAPKAPK2*	0.0022	0.0488
	hsa05142: Chagas disease (American trypanosomiasis)	*AKT2, MYD88, ADCY1, TLR4, CFLAR, PIK3CD, IKBKG, IL1B, MAPK3, TGFB1, CCL2*	0.0024	0.0488
	hsa05140: leishmaniasis	*ELK1, ITGB2, PTPN6, MYD88, NFKBIB, TLR4, IL1B, MAPK3, TGFB1*	0.0024	0.0488
	hsa04668: TNF signaling pathway	*AKT2, CREB5, MAP3K5, CFLAR, PIK3CD, IKBKG, TNFRSF1B, IL1B, MAPK3, CCL2, JUNB*	0.0029	0.0547
	hsa05161: hepatitis B	*AKT2, CREB5, ELK1, IKBKG, MAPK3, CDKN1A, BAX, MYD88, TLR4, RAF1, PIK3CD, STAT3, TGFB1*	0.0033	0.0553

#### Unique Genes for MBPBs in Analyzed Models

We compared the lists of genes in altered KEGG pathways (Supplementary excel file_1 - Most Frequent Genes in Pathways). We found that five downregulated genes (*STAT3*, *CDKN1A*, *PDGFB*, *SHC1*, and *ELK1*) were most frequently repeated in the KEGG pathways unique for agomelatine treatment.

## Discussion

### Genes and Polymorphisms

In the present study, we analyzed genes involved in the MEL metabolism pathway and selected polymorphisms associated with symptoms of mental disorders (Dmitrzak-Weglarz and Reszka, 2017). The studies of polymorphisms in the MEL pathway genes are few, ambiguous, and outdated (Kripke et al., 2011). Only in rs4446909 *ASMT* is the association confirmed with UD, BD, reduced *ASMT* mRNA expression, and lower enzymatic activity of ASMT (Etain et al., 2012; Geoffroy et al., 2014). Our study found an association between two polymorphisms (rs8150 and rs3760138) in the *AANAT* gene and bipolar depression. We also observed that variants of the rs3760138 polymorphism correlate with the concentration of MEL measured in the blood serum of patients experiencing aggravation of depressive symptoms and the concentration of the enzyme itself in remission. The results of our research are consistent with those of the analysis presented by Soria et al. (2010). We discovered that the TT variant genotype is associated with both the risk of depression and a higher concentration of the encoded enzyme. Interestingly, in a case–control study of Norwegian nurses working night shifts, the GG carriers of rs3760138 in the *AANAT* gene showed an increased risk of breast cancer (Zienolddiny et al., 2013). The present results provide evidence that *AANAT* gene variants play a role in the pathophysiology of depression and support the hypothesis of the MEL signaling pathway in depression. The lack of significant correlations in the remaining polymorphisms may result from a weak association that is elusive in a limited clinical trial and does not exclude the influence of these genes in the metabolism of MEL and the depressive disorders related to it. The overall evidence from our study and other studies encourages the continuation of more detailed analyses of this pathway.

### Serum Biomarkers

Measurements of the four critical biomarker concentrations in the MEL pathway in the blood serum confirmed the disturbances of this pathway in depressive disorders and their partial normalization after treatment. In both UD and BD, patients revealed different patterns of concentration of individual biomarkers before and after treatment and compared to the control group.

Our study found that the concentration of global serotonin in the blood serum was decreased nominally in UD and statistically significantly in BD. These results are consistent with the serotonin concentration measurements in the plasma of patients with depression (Saldanha et al., 2009). It is indicated that due to the rapid mitochondrial metabolism of serotonin by the MAO enzyme to 5-hydroxyindole acetic acid (5HIAA), the latter forms a better biomarker of SERT biotransformation (Jayamohananan et al., 2019). A significantly higher concentration of 5HIAA in the plasma of patients with depression suggests a rapid breakdown of serotonin in the brain (T P et al., 2008). The serotonin hypothesis of depression indicates that impaired serotonin function may cause clinical depression, but it is unnecessary or sufficient (Cowen and Browning, 2015).

We observed that the concentrations of AANAT and ASMT enzymes were nominally elevated in UD and statistically significantly increased in BD. These results identify and confirm changes in the expression level of the enzymes depending on the type of depression, although earlier in depression (Talarowska et al., 2014) and the autism spectrum, the decreased activity of these enzymes was demonstrated (Pagan et al., 2017). Of particular interest was the increase in the concentration of both the AANAT and ASMT enzymes observed in UD after treatment. Antidepressants, in particular SSRIs, inhibit serotonin reuptake by increasing its concentration in the inter-synaptic space, which leads to increased stimulation of the postsynaptic serotonin receptors.

The expression of enzymes that use serotonin to synthesize MEL can potentially be stimulated by pharmacologically disturbed serotonin levels. On the other hand, faster serotonin turnover may prevent the capturing of subtle changes in peripheral serotonin concentration (Stapelberg et al., 2018).

In our study, the MEL concentration in the blood serum was significantly lower in patients with depression regardless of the type of depression, which is consistent with previous reports (Khaleghipour et al., 2012; Oglodek et al., 2016). This observation also partially overlaps with the results of Bumb et al. (2016). Additionally, they also confirmed altered MEL levels in depressed patients. Like us, they selected a group of patients with UD and BD and also measured the MEL concentration in the cerebrospinal fluid. In the serum, they observed a significant decrease in MEL for UD but not for BD, while we observed a decrease in both. The discrepancies in the results may be due to our inclusion of a homogeneous BD group only in the depressive state, while Bumb et al. included patients in manic and depressive states. On the other hand, in the CSF, they observed a significantly reduced MEL level in BD, unlike in the serum, but not in UD. Our study, in turn, is in contradiction with the results of the Chinese population. The serum MEL levels in the first-episode and recurrent groups were significantly higher than those in the control group. The authors stated that the results may have been caused by the measurement technique used, the treatment applied, and the stratification effect (Tao et al., 2020). We have observed that MEL secretion disturbance is more severe in BD and more challenging to normalize with treatment. Persistent disturbance of MEL may be associated with the maintenance of sleep disorders (mainly reduced need for sleep), and these, in turn, contribute to more frequent relapses and greater intensity of depression symptoms, which we observed in the studied BD group. The results of our research provide molecular evidence for the validity of the methods used for the regulation and normalization of sleep and wake rhythm in BD therapy (Miklowitz et al., 2012; Alston et al., 2019).

To summarize, the results suggest the existence of diverse, specific patterns of serum concentration of MBPBs for types of depression, which do not exclude the existence of tissue-specific patterns. Longitudinal studies are needed to investigate the phase–disease relationship in MEL changes and the causes and consequences of site-specific changes.

### Clinical Model

We performed a screening analysis of gene expression profiling in PBMC obtained from patients hospitalized due to a depressive episode to detect altered genes in patients treated with agomelatine. Agomelatine is a synthetic analog of MEL and acts as an agonist on the MT1 and MT2 receptors. Agomelatine also acts as an antagonist on the 5-HT2C receptors and has a low affinity for most other receptors, including adrenoceptors, dopamine, GABA, muscarinic, histamine, benzodiazepine, and sigma receptors, as well as ion channels (Norman and Olver, 2019). We selected a list of genes specific to agomelatine action that formed twelve significant downregulated pathways. Interaction analyses showed that approximately 60 proteins of these pathways had interacted together.

The common denominator of the identified pathways was participation in inflammatory processes (i.e., chemokines, neurotrophins, and cytokines). In particular, neurotrophic factors regulate the survival, development, and function of nervous tissue. Inhibition of inflammation is essential for neuroprotection and antiapoptotic activities (Skaper, 2018). We know that MEL acts as a pro-inflammatory modulator when the body is attacked by pathogens (Hardeland et al., 2015). However, when the inflammation increases to the extent that there is a risk of cell damage, it becomes an anti-inflammatory modulator (Carrillo-Vico et al., 2013). In this way, it protects the body against the development of chronic inflammatory diseases (Radogna et al., 2010). The results confirmed that agomelatine, as an analog of MEL, may act as a "buffer" of the immune system and improve depressive symptoms. Thus, attempts have been made to use exogenous MEL to treat COVID-19 caused by the SARS-CoV-2 virus, especially in the elderly with a reduced level of MEL (Kleszczynski et al., 2020; Shneider et al., 2020).

Melatonin and its ability to modulate inflammation in multiple sclerosis have been the subject of intense research in recent years. The high coexistence of depression is also observed in this disease with one common denominator—immunological changes (Feinstein et al., 2014; Solaro et al., 2018). Multiple sclerosis (MS) is an inflammatory demyelinating disease. An increased risk of this disease has been observed in residents in high-latitude countries where sunlight is limited and populations are deficient in vitamin D and high levels of melatonin (Ghareghani et al., 2018). It has been suggested that oxidative stress could be responsible for this disease. Thus, melatonin, as a natural antioxidant in the CNS, may constitute helpful therapy. Ghareghani et al. (2018), using a mouse model of MS, showed that melatonin promoted remyelination, decreased inflammation, and stimulated the activity of antioxidant enzymes (Ghareghani et al., 2017; Ghareghani et al., 2018). The research team also showed that MEL administration in the acute phase of MS might exacerbate encephalomyelitis in line with the aforementioned dualistic MEL function as a pro- or anti-inflammatory modulator depending on inflammation intensity (Ghareghani et al., 2017). In MS treatment, a significant impact is caused by time and dose of MEL and bilateral interaction between used drugs. For example, MEL improved the baclofen effect on spasticity (Ghareghani et al., 2018). In turn, corticosteroids decreased MEL levels and caused sleep disturbance (Dokoohaki et al., 2017).

The analysis of the most frequently repeated genes in the identified pathways, which changed explicitly under the influence of agomelatine, revealed the following genes:

*STAT3* (signal transducer and activator of transcription 3) acts as a transcription activator in response to cytokines and growth factors
*CDKN1A* (cyclin-dependent kinase inhibitor 1A) functions as a regulator of cell cycle progression at G1
*PDGFB* (platelet-derived growth factor subunit B) activates PDGF receptor tyrosine kinases, which play a role in a wide range of developmental processes
*SHC1* (SHC adaptor protein 1) is an adapter protein in the signal transduction pathways
*ELK1* (ETS transcription factor ELK1) is a transcription factor that binds to purine-rich DNA sequences and induces target gene transcription upon JNK-signaling pathway stimulation.

The genes listed above belong to the transcription factor group that influences gene expression regulation and, thus, the etiology of many mental disorders or symptoms. Therefore, their further detailed study is necessary (Semenza, 2005; Fuxman Bass et al., 2015; Papavassiliou and Papavassiliou, 2016).

### Cell Culture Model

To check the changes taking place in the brain under the influence of MEL, we conducted a pilot experiment using cell cultures based on mouse hippocampal neurons. We identified ten downregulated pathways (hsa03018: RNA degradation, hsa05200: pathways in cancer, hsa03460: Fanconi anemia pathway, hsa01100: metabolic pathways, hsa04713: circadian entrainment, hsa01130: biosynthesis of antibiotics, hsa05134: legionellosis, hsa04810: regulation of actin cytoskeleton, hsa04726: serotonergic synapse, and hsa04610: complement and coagulation cascades). These results demonstrate the influence of MEL in the CNS on the generally understood neuronal metabolism, including the serotoninergic system and circadian rhythms. Additionally, they indicate the participation of disturbances of the MEL level in the etiology of depression.

Wiechmann (2002), for the first time, presented the results of DEG analyses in two types of MEL-treated rat eye nerve cells using the microarray technique. Although identified, the genes with altered expression in both types of examined cells did not coincide, indicating not only the tissue but also the cellular specificity of MEL action (Wiechmann, 2002).

In turn, Ha et al. (2006), for the first time, presented the results of the microarray analysis of MEL-treated PBMC cells obtained from healthy volunteers. Forty-six upregulated and twenty-three downregulated genes were identified. Most genes formed a cellular physiological response pathway. In this pathway, the most remarkable upregulation change was shown by the *CFTR* gene (cystic fibrosis transmembrane conductance regulator) and the downregulation by gene *UBE2M* (ubiquitin-conjugating enzyme E2M) (Ha et al., 2006). In 2007, the same team performed a microarray analysis with a modified protocol (Park et al., 2007). MEL reduced the expression of genes associated with inflammation. In particular, chemokine genes were downregulated, which we also observed in patients taking agomelatine. These results confirmed that MEL exerts anti-inflammatory properties by regulating the chemokines, which act as crucial mediators in various inflammatory processes (Ban et al., 2011).

In the cell culture model under study, we observed a relatively large number of downregulated genes. The distribution of melatonin receptors in the hippocampal cells is lower than that of the cerebellum or occipital cortex (Mazzucchelli et al., 1996; Pandiperumal et al., 2008; Klosen et al., 2019). Despite the selection of the experimental dose of melatonin based on the available literature data, we cannot exclude the possibility that the unbound melatonin exerts a subtoxic effect on NH cells, expressed by an increased number of downexpressed genes.

To summarize, we obtained a joint conclusion confirming that MEL is involved in modulating the immune system. The effect on the expression of the number and type of genes depends on the model tested (human/mouse), type of cells tested (PBMC/macrophages), protocol (incubation time/concentrations of mediators used), and type of microarray (global/dedicated). Hence, it is not easy to make a detailed comparison, as each of the discussed studies showed different sets of genes either up- or down-regulated by MEL.

### Limitations

Our screening microarray results include female patients only, so the study should be repeated in an independent and homogeneous group of men. The expression data obtained from PBMC reflect only about 85% of the changes in the brain, disregarding the tissue-specific ones. Measurement of the general SERT and MEL levels in serum may not be the optimal method. Although the NH cell line is currently the best research model for cell pathways in neuroscience (Seibenhener and Wooten, 2012), it is not necessarily an equally good model for observing melatonin effects. Compared to other brain structures, the hippocampus has a lower expression of melatonin receptors (Mazzucchelli et al., 1996; Klosen et al., 2019). Finally, the total sample size was small, although comparable to other available studies, and achieved 80% power.

## Conclusion

The microarray analysis results should be treated as preliminary because they are not without limitations, mainly of a small test sample. However, they are a valuable complement to clinical observations at the molecular level consistent in the modulation of the immune system by melatonin. These results may help determine further research directions, such as searching for more adequate cell models for the melatonin pathway or functional research focused on transcription factors that are challenging to interpret due to multivariate determinants. It may also be essential to develop a uniform pipeline of bioinformatic analyses in the future (critical cutoff thresholds, applied corrections, and algorithms to predict gene ontology annotations), which currently may give partially different or complementary results in different centers.

In summary, we confirm the dysfunctions in the molecular regulation of the MEL biosynthesis pathway with a specific pattern for unipolar and bipolar depression at the tier of genes, their polymorphisms, and examined serum biomarkers. Using a systematic search of DEGs with restricted thresholds, we confirm that microarray profiling is a useful biological marker search tool. The biological pathway analysis uncovered pathways and genes uniquely altered after agomelatine treatment in a clinical sample and MEL treatment in cell culture. In both models, we confirmed the immunomodulatory effect of MEL agents in depression.

## Data Availability

The datasets presented in this study can be found in online repositories. The names of the repository/repositories and accession number(s) can be found below: NCBI Gene Expression Omnibus, accession no: GSE169459 (Edgar et al., 2002).

## Footnotes

1
https://biosearch-cdn.azureedge.net/assetsv6/KASP-genotyping-chemistry-User-guide.pdf

2
https://biosearch-cdn.azureedge.net/assetsv6/Analysis-of-KASP-genotyping-data-using-cluster-plots.pdf

3
https://biosearch-cdn.azureedge.net/assetsv6/KASP-thermal-cycling-61-55%C2%BAC-touchdown-protocol.pdf

4
https://www.thermofisher.com/pl/en/home/references/protocols/nucleic-acid-purification-and-analysis/rna-protocol/alternative-protocol-extraction-of-rna-from-cells-captured-on-leukolock-filters-using-tri-reagent.html

## References

[B1] Alston M. Cain S. W. Rajaratnam S. M. W. (2019). Advances of Melatonin-Based Therapies in the Treatment of Disturbed Sleep and Mood. Handb Exp. Pharmacol. 253, 305–319. 10.1007/164_2018_139 31123831

[B2] Baldessarini R. J. Tondo L. Vázquez G. H. (2019). Pharmacological Treatment of Adult Bipolar Disorder. Mol. Psychiatry 24 (2), 198–217. 10.1038/s41380-018-0044-2 29679069

[B3] Ban J. Y. Kim B. S. Kim S. C. Kim D. H. Chung J.-H. (2011). Microarray Analysis of Gene Expression Profiles in Response to Treatment with Melatonin in Lipopolysaccharide Activated RAW 264.7 Cells. Korean J. Physiol. Pharmacol. 15 (1), 23–29. 10.4196/kjpp.2011.15.1.23 21461237 PMC3062080

[B4] Boyce P. Hopwood M. (2013). Manipulating Melatonin in Managing Mood. Acta Psychiatr. Scand. 128 (444), 16–23. 10.1111/acps.12175 23909693

[B5] Bumb J. M. Enning F. Mueller J. K. van der List T. Rohleder C. Findeisen P. (2016). Differential Melatonin Alterations in Cerebrospinal Fluid and Serum of Patients with Major Depressive Disorder and Bipolar Disorder. Compr. Psychiatry 68, 34–39. 10.1016/j.comppsych.2016.03.005 27234180

[B6] Carman J. S. Post R. M. Buswell R. Goodwin F. K. (1976). Negative Effects of Melatonin on Depression. Am. J. Psychiatry 133 (10), 1181–1186. 10.1176/ajp.133.10.1181 788529

[B7] Carrillo-Vico A. Lardone P. Álvarez-Sánchez N. Rodríguez-Rodríguez A. Guerrero J. (2013). Melatonin: Buffering the Immune System. Ijms 14 (4), 8638–8683. 10.3390/ijms14048638 23609496 PMC3645767

[B8] Cheng C. Pounds S. (2007). False Discovery Rate Paradigms for Statistical Analyses of Microarray Gene Expression Data. Bioinformation 1 (10), 436–446. 10.6026/97320630001436 17597936 PMC1896060

[B9] Cowen P. J. Browning M. (2015). What Has Serotonin to Do with Depression? World Psychiatry 14 (2), 158–160. 10.1002/wps.20229 26043325 PMC4471964

[B10] Dalman M. R. Deeter A. Nimishakavi G. Duan Z.-H. (2012). Fold Change and P-Value Cutoffs Significantly Alter Microarray Interpretations. BMC Bioinformatics 13 (Suppl. 2), S11. 10.1186/1471-2105-13-S2-S11 PMC3305783 22536862

[B12] De Berardis D. Fornaro M. Serroni N. Campanella D. Rapini G. Olivieri L. (2015). Agomelatine beyond Borders: Current Evidences of its Efficacy in Disorders Other Than Major Depression. Ijms 16 (1), 1111–1130. 10.3390/ijms16011111 25569089 PMC4307293

[B11] De Berardis D. Marini S. Fornaro M. Srinivasan V. Iasevoli F. Tomasetti C. (2013). The Melatonergic System in Mood and Anxiety Disorders and the Role of Agomelatine: Implications for Clinical Practice. Ijms 14 (6), 12458–12483. 10.3390/ijms140612458 23765220 PMC3709794

[B16] Dmitrzak-Weglarz M. Pawlak J. Wilkosc M. Miechowicz I. Maciukiewicz M. Ciarkowska W. (2016). Chronotype and Sleep Quality as a Subphenotype in Association Studies of Clock Genes in Mood Disorders. Acta Neurobiol. Exp. (Wars) 76 (1), 32–42. 10.21307/ane-2017-003 27102916

[B15] Dmitrzak-Weglarz M. P. Pawlak J. M. Maciukiewicz M. Moczko J. Wilkosc M. Leszczynska-Rodziewicz A. (2015). Clock Gene Variants Differentiate Mood Disorders. Mol. Biol. Rep. 42 (1), 277–288. 10.1007/s11033-014-3770-9 25258123

[B14] Dmitrzak-Weglarz M. Reszka E. (2017). Pathophysiology of Depression: Molecular Regulation of Melatonin Homeostasis - Current Status. Neuropsychobiology 76 (3), 117–129. 10.1159/000489470 29898451

[B17] Dokoohaki S. Ghareghani M. Ghanbari A. Farhadi N. Zibara K. Sadeghi H. (2017). Corticosteroid Therapy Exacerbates the Reduction of Melatonin in Multiple Sclerosis. Steroids 128, 32–36. 10.1016/j.steroids.2017.10.006 29061489

[B18] Edgar R. Domrachev M. Lash A. E. (2002). Gene Expression Omnibus: NCBI Gene Expression and Hybridization Array Data Repository. Nucleic Acids Res. 30 (1), 207–210. 10.1093/nar/30.1.207 11752295 PMC99122

[B19] Etain B. Dumaine A. Bellivier F. Pagan C. Francelle L. Goubran-Botros H. (2012). Genetic and Functional Abnormalities of the Melatonin Biosynthesis Pathway in Patients with Bipolar Disorder. Hum. Mol. Genet. 21 (18), 4030–4037. 10.1093/hmg/dds227 22694957

[B21] Faul F. Erdfelder E. Buchner A. Lang A.-G. (2009). Statistical Power Analyses Using G*Power 3.1: Tests for Correlation and Regression Analyses. Behav. Res. Methods 41 (4), 1149–1160. 10.3758/BRM.41.4.1149 19897823

[B20] Faul F. Erdfelder E. Lang A.-G. Buchner A. (2007). G*Power 3: A Flexible Statistical Power Analysis Program for the Social, Behavioral, and Biomedical Sciences. Behav. Res. Methods 39 (2), 175–191. 10.3758/bf03193146 17695343

[B22] Feinstein A. Magalhaes S. Richard J.-F. Audet B. Moore C. (2014). The Link between Multiple Sclerosis and Depression. Nat. Rev. Neurol. 10 (9), 507–517. 10.1038/nrneurol.2014.139 25112509

[B23] First M. B. Spitzer R. L. Gibbon M. Williams J. (1996). Structured Clinical Interview for DSM‐IV Axis I Disorders, Clinician Version (SCID‐CV). Washington, D.C.: American Psychiatric Press.

[B25] Fornaro M. Anastasia A. Monaco F. Novello S. Fusco A. Iasevoli F. (2018). Clinical and Psychopathological Features Associated with Treatment-Emergent Mania in Bipolar-II Depressed Outpatients Exposed to Antidepressants. J. Affect. Disord. 234, 131–138. 10.1016/j.jad.2018.02.085 29525354

[B24] Fornaro M. Bandini F. Cestari L. Cordano C. Ogliastro C. Albano C. (2014). Electroretinographic Modifications Induced by Agomelatine: a Novel Avenue to the Understanding of the Claimed Antidepressant Effect of the Drug? Ndt 10, 907–914. 10.2147/NDT.S63459 PMC4038423 24899809

[B26] Forty L. Smith D. Jones L. Jones I. Caesar S. Cooper C. (2008). Clinical Differences between Bipolar and Unipolar Depression. Br. J. Psychiatry 192 (5), 388–389. 10.1192/bjp.bp.107.045294 18450667

[B27] Fuxman Bass J. I. Sahni N. Shrestha S. Garcia-Gonzalez A. Mori A. Bhat N. (2015). Human Gene-Centered Transcription Factor Networks for Enhancers and Disease Variants. Cell 161 (3), 661–673. 10.1016/j.cell.2015.03.003 25910213 PMC4409666

[B28] Gautam A. Donohue D. Hoke A. Miller S. A. Srinivasan S. Sowe B. (2019). Investigating Gene Expression Profiles of Whole Blood and Peripheral Blood Mononuclear Cells Using Multiple Collection and Processing Methods. PLoS One 14 (12), e0225137. 10.1371/journal.pone.0225137 31809517 PMC6897427

[B29] Geoffroy P. A. Boudebesse C. Henrion A. Jamain S. Henry C. Leboyer M. (2014). An ASMT Variant Associated with Bipolar Disorder Influences Sleep and Circadian Rhythms: a Pilot Study. Genes, Brain Behav. 13 (3), 299–304. 10.1111/gbb.12103 24308489

[B30] Germain A. Kupfer D. J. (2008). Circadian Rhythm Disturbances in Depression. Hum. Psychopharmacol. Clin. Exp. 23 (7), 571–585. 10.1002/hup.964 PMC2612129 18680211

[B31] Ghareghani M. Dokoohaki S. Ghanbari A. Farhadi N. Zibara K. Khodadoust S. (2017). Melatonin Exacerbates Acute Experimental Autoimmune Encephalomyelitis by Enhancing the Serum Levels of Lactate: A Potential Biomarker of Multiple Sclerosis Progression. Clin. Exp. Pharmacol. Physiol. 44 (1), 52–61. 10.1111/1440-1681.12678 27696474

[B33] Ghareghani M. Reiter R. J. Zibara K. Farhadi N. (2018). Latitude, Vitamin D, Melatonin, and Gut Microbiota Act in Concert to Initiate Multiple Sclerosis: A New Mechanistic Pathway. Front. Immunol. 9, 2484. 10.3389/fimmu.2018.02484 30459766 PMC6232868

[B32] Ghareghani M. Sadeghi H. Zibara K. Danaei N. Azari H. Ghanbari A. (2017). Melatonin Increases Oligodendrocyte Differentiation in Cultured Neural Stem Cells. Cell Mol. Neurobiol. 37 (7), 1319–1324. 10.1007/s10571-016-0450-4 27987059 PMC11482234

[B34] Ghareghani M. Zibara K. Sadeghi H. Farhadi N. (2018). Spasticity Treatment Ameliorates the Efficacy of Melatonin Therapy in Experimental Autoimmune Encephalomyelitis (EAE) Mouse Model of Multiple Sclerosis. Cell Mol. Neurobiol. 38 (5), 1145–1151. 10.1007/s10571-018-0580-y 29497878 PMC11481852

[B35] Ha E. Han E. Park H. J. Kim H.-J. Hong M. S. Hong S.-J. (2006). Microarray Analysis of Transcription Factor Gene Expression in Melatonin-Treated Human Peripheral Blood Mononuclear Cells. J. Pineal Res. 40 (4), 305–311. 10.1111/j.1600-079X.2006.00317.x 16635017

[B36] Hamilton M. (1960). A Rating Scale for Depression. J. Neurol. Neurosurg. Psychiatry 23, 56–62. 10.1136/jnnp.23.1.56 14399272 PMC495331

[B37] Han K.-M. De Berardis D. Fornaro M. Kim Y.-K. (2019). Differentiating between Bipolar and Unipolar Depression in Functional and Structural MRI Studies. Prog. Neuro-Psychopharmacol. Biol. Psychiatry 91, 20–27. 10.1016/j.pnpbp.2018.03.022 29601896

[B38] Hardeland R. Cardinali D. P. Brown G. M. Pandi-Perumal S. R. (2015). Melatonin and Brain Inflammaging. Prog. Neurobiol. 127–128, 46–63. 10.1016/j.pneurobio.2015.02.001 25697044

[B39] Hickie I. B. Rogers N. L. (2011). Novel Melatonin-Based Therapies: Potential Advances in the Treatment of Major Depression. The Lancet 378 (9791), 621–631. 10.1016/S0140-6736(11)60095-0 21596429

[B40] Huang D. W. Sherman B. T. Lempicki R. A. (2009). Systematic and Integrative Analysis of Large Gene Lists Using DAVID Bioinformatics Resources. Nat. Protoc. 4 (1), 44–57. 10.1038/nprot.2008.211 19131956

[B41] Iasevoli F. Valchera A. Di Giovambattista E. Marconi M. Rapagnani M. P. De Berardis D. (2013). Affective Temperaments Are Associated with Specific Clusters of Symptoms and Psychopathology: a Cross-Sectional Study on Bipolar Disorder Inpatients in Acute Manic, Mixed, or Depressive Relapse. J. Affective Disord. 151 (2), 540–550. 10.1016/j.jad.2013.06.041 23856282

[B42] Jayamohananan H. Manoj Kumar M. K. Aneesh T. P. (2019). 5-HIAA as a Potential Biological Marker for Neurological and Psychiatric Disorders. Adv. Pharm. Bull. 9 (3), 374–381. 10.15171/apb.2019.044 31592064 PMC6773935

[B43] Kanehisa M. Sato Y. Furumichi M. Morishima K. Tanabe M. (2019). New Approach for Understanding Genome Variations in KEGG. Nucl. Acids Res. 47 (D1), D590–D595. 10.1093/nar/gky962 30321428 PMC6324070

[B44] Khaleghipour S. Masjedi M. Ahade H. Enayate M. Pasha G. Nadery F. (2012). Morning and Nocturnal Serum Melatonin Rhythm Levels in Patients with Major Depressive Disorder: an Analytical Cross-Sectional Study. Sao Paulo Med. J. 130 (3), 167–172. 10.1590/s1516-31802012000300006 22790549 PMC10876199

[B45] Kleber A. Ruf C. G. Wolf A. Fink T. Glas M. Wolf B. (2015). Melatonin or Ramelteon Therapy Differentially Affects Hepatic Gene Expression Profiles after Haemorrhagic Shock in Rat - A Microarray Analysis. Exp. Mol. Pathol. 99 (2), 189–197. 10.1016/j.yexmp.2015.06.019 26116814

[B46] Kleszczynski K. Slominski A. T. Steinbrink K. Reiter R. J. (2020). Clinical Trials for Use of Melatonin to Fight against COVID-19 Are Urgently Needed. Nutrients 12 (9), 2561. 10.3390/nu12092561 PMC7551551 32847033

[B47] Klosen P. Lapmanee S. Schuster C. Guardiola B. Hicks D. Pevet P. (2019). MT1 and MT2 Melatonin Receptors Are Expressed in Nonoverlapping Neuronal Populations. J. Pineal Res. 67 (1), e12575. 10.1111/jpi.12575 30937953

[B48] Konstantakopoulos G. Dimitrakopoulos S. Michalopoulou P. G. (2020). The Preclinical Discovery and Development of Agomelatine for the Treatment of Depression. Expert Opin. Drug Discov. 15 (10), 1121–1132. 10.1080/17460441.2020.1781087 32568567

[B49] Kripke D. F. Nievergelt C. M. Tranah G. J. Murray S. S. McCarthy M. J. Rex K. M. (2011). Polymorphisms in Melatonin Synthesis Pathways: Possible Influences on Depression. J. Circadian Rhythms 9, 8. 10.1186/1740-3391-9-8 21827647 PMC3177871

[B50] Lecrubier Y. Sheehan D. Weiller E. Amorim P. Bonora I. Sheehan K. H. (1997). The Mini International Neuropsychiatric Interview (MINI). A Short Diagnostic Structured Interview: Reliability and Validity According to the CIDI. Eur. Psychiatr. 12 (5), 224–231. 10.1016/s0924-9338(97)83296-8

[B51] Mahmood D. (2019). Pleiotropic Effects of Melatonin. Drug Res. (Stuttg) 69 (2), 65–74. 10.1055/a-0656-6643 30060265

[B52] Malhi G. S. Kuiper S. (2013). Chronobiology of Mood Disorders. Acta Psychiatr. Scand. 128 (444), 2–15. 10.1111/acps.12173 23909692

[B53] Mazzucchelli C. Pannacci M. Nonno R. Lucini V. Fraschini F. Stankov B. M. (1996). The Melatonin Receptor in the Human Brain: Cloning Experiments and Distribution Studies. Brain Res. Mol. Brain Res. 39 (1-2), 117–126. 10.1016/0169-328x(96)00017-4 8804720

[B54] McGuffin P. Farmer A. Harvey I. (1991). A Polydiagnostic Application of Operational Criteria in Studies of Psychotic Illness. Arch. Gen. Psychiatry 48 (8), 764–770. 10.1001/archpsyc.1991.01810320088015 1883262

[B55] Melo M. C. A. Abreu R. L. C. Linhares Neto V. B. de Bruin P. F. C. de Bruin V. M. S. (2017). Chronotype and Circadian Rhythm in Bipolar Disorder: A Systematic Review. Sleep Med. Rev. 34, 46–58. 10.1016/j.smrv.2016.06.007 27524206

[B56] Mendoza J. (2019). Circadian Insights into the Biology of Depression: Symptoms, Treatments and Animal Models. Behav. Brain Res. 376, 112186. 10.1016/j.bbr.2019.112186 31473283

[B57] Miklowitz D. J. Price J. Holmes E. A. Rendell J. Bell S. Budge K. (2012). Facilitated Integrated Mood Management for Adults with Bipolar Disorder. Bipolar Disord. 14 (2), 185–197. 10.1111/j.1399-5618.2012.00998.x 22420594 PMC3412076

[B58] Miller S. A. Dykes D. D. Polesky H. F. (1988). A Simple Salting Out Procedure for Extracting DNA from Human Nucleated Cells. Nucl. Acids Res. 16 (3), 1215. 10.1093/nar/16.3.1215 3344216 PMC334765

[B59] Norman T. R. Olver J. S. (2019). Agomelatine for Depression: Expanding the Horizons? Expert Opin. Pharmacother. 20 (6), 647–656. 10.1080/14656566.2019.1574747 30759026

[B60] Oglodek E. A. Just M. J. Szromek A. R. Araszkiewicz A. (2016). Melatonin and Neurotrophins NT-3, BDNF, NGF in Patients with Varying Levels of Depression Severity. Pharmacol. Rep. 68 (5), 945–951. 10.1016/j.pharep.2016.04.003 27367919

[B61] Pagan C. Goubran-Botros H. Delorme R. Benabou M. Lemière N. Murray K. (2017). Disruption of Melatonin Synthesis Is Associated with Impaired 14-3-3 and miR-451 Levels in Patients with Autism Spectrum Disorders. Sci. Rep. 7 (1), 2096. 10.1038/s41598-017-02152-x 28522826 PMC5437096

[B62] Palazidou E. Papadopoulos A. Ratcliff H. Dawling S. Checkley S. A. (1992). Noradrenaline Uptake Inhibition Increases Melatonin Secretion, a Measure of Noradrenergic Neurotransmission, in Depressed Patients. Psychol. Med. 22 (2), 309–315. 10.1017/s0033291700030257 1319597

[B63] Pandiperumal S. Trakht I. Srinivasan V. Spence D. Maestroni G. Zisapel N. (2008). Physiological Effects of Melatonin: Role of Melatonin Receptors and Signal Transduction Pathways. Prog. Neurobiol. 85 (3), 335–353. 10.1016/j.pneurobio.2008.04.001 18571301

[B64] Papavassiliou K. A. Papavassiliou A. G. (2016). Transcription Factor Drug Targets. J. Cel. Biochem. 117 (12), 2693–2696. 10.1002/jcb.25605 27191703

[B65] Park H. J. Kim H. J. Ra J. Hong S.-J. Baik H. H. Park H.-K. (2007). Melatonin Inhibits Lipopolysaccharide-Induced CC Chemokine Subfamily Gene Expression in Human Peripheral Blood Mononuclear Cells in a Microarray Analysis. J. Pineal Res. 43 (2), 121–129. 10.1111/j.1600-079X.2007.00452.x 17645690

[B66] Radogna F. Diederich M. Ghibelli L. (2010). Melatonin: a Pleiotropic Molecule Regulating Inflammation. Biochem. Pharmacol. 80 (12), 1844–1852. 10.1016/j.bcp.2010.07.041 20696138

[B67] Rybakowski J. K. (2018). Meaningful Aspects of the Term 'mood Stabilizer'. Bipolar Disord. 20 (4), 391–392. 10.1111/bdi.12608 29327794

[B68] Saldanha D. Kumar N. Ryali V. Srivastava K. Pawar A. (2009). Serum Serotonin Abnormality in Depression. Med. J. Armed Forces India 65 (2), 108–112. 10.1016/S0377-1237(09)80120-2 27408213 PMC4921409

[B69] Salva M. A. Q. Hartley S. (2012). Mood Disorders, Circadian Rhythms, Melatonin and Melatonin Agonists. J. Cent. Nerv Syst. Dis. 4, JCNSD.S4103–26. 10.4137/JCNSD.S4103 PMC3619438 23650464

[B70] Schroeder A. Mueller O. Stocker S. Salowsky R. Leiber M. Gassmann M. (2006). The RIN: an RNA Integrity Number for Assigning Integrity Values to RNA Measurements. BMC Mol. Biol. 7, 3. 10.1186/1471-2199-7-3 16448564 PMC1413964

[B71] Seibenhener M. L. Wooten M. W. (2012). Isolation and Culture of Hippocampal Neurons from Prenatal Mice. JoVE 65, 3634. 10.3791/3634 PMC3476399 22871921

[B72] Semenza G. L. (2005). Transcription Factors and Human Disorders. In: Encyclopedia of Life Sciences. New York City: John Wiley & Sons. 10.1038/npg.els.0005504

[B73] Serretti A. (2017). Genetics and Pharmacogenetics of Mood Disorders. Psychiatr. Pol. 51 (2), 197–203. 10.12740/PP/68914 28581531

[B74] Shneider A. Kudriavtsev A. Vakhrusheva A. (2020). Can Melatonin Reduce the Severity of COVID-19 Pandemic? Int. Rev. Immunol. 39 (4), 153–162. 10.1080/08830185.2020.1756284 32347747

[B75] Singh M. Jadhav H. R. (2014). Melatonin: Functions and Ligands. Drug Discov. Today 19 (9), 1410–1418. 10.1016/j.drudis.2014.04.014 24792719

[B76] Skaper S. D. (2018). Neurotrophic Factors: An Overview. Methods Mol. Biol. 1727, 1–17. 10.1007/978-1-4939-7571-6_1 29222769

[B77] Solaro C. Gamberini G. Masuccio F. G. (2018). Depression in Multiple Sclerosis: Epidemiology, Aetiology, Diagnosis and Treatment. CNS Drugs 32 (2), 117–133. 10.1007/s40263-018-0489-5 29417493

[B78] Soria V. Martínez-Amorós È. Escaramís G. Valero J. Crespo J. M. Gutiérrez-Zotes A. (2010). Resequencing and Association Analysis of Arylalkylamine N-Acetyltransferase (AANAT) Gene and its Contribution to Major Depression Susceptibility. J. Pineal Res. 49 (1), no. 10.1111/j.1600-079X.2010.00763.x 20459461

[B79] Stapelberg N. J. C. Pratt R. Neumann D. L. Shum D. H. K. Brandis S. Muthukkumarasamy V. (2018). From Feedback Loop Transitions to Biomarkers in the Psycho-Immune-Neuroendocrine Network: Detecting the Critical Transition from Health to Major Depression. Neurosci. Biobehav. Rev. 90, 1–15. 10.1016/j.neubiorev.2018.03.005 29524456

[B80] Szklarczyk D. Gable A. L. Lyon D. Junge A. Wyder S. Huerta-Cepas J. (2019). STRING V11: Protein-Protein Association Networks with Increased Coverage, Supporting Functional Discovery in Genome-wide Experimental Datasets. Nucl. Acids Res. 47 (D1), D607–D613. 10.1093/nar/gky1131 30476243 PMC6323986

[B81] T P D. A. Esler M. D. Dawood T. Lambert E. A. Haikerwal D. Brenchley C. (2008). Elevated Brain Serotonin Turnover in Patients with Depression. Arch. Gen. Psychiatry 65 (1), 38–46. 10.1001/archgenpsychiatry.2007.11 18180427

[B82] Talarowska M. Szemraj J. Zajaczkowska M. Galecki P. (2014). ASMT Gene Expression Correlates with Cognitive Impairment in Patients with Recurrent Depressive Disorder. Med. Sci. Monit. 20, 905–912. 10.12659/MSM.890160 24881886 PMC4052942

[B83] Tao H. Chen X. Zhou H. Fu J. Yu Q. Liu Y. (2020). Changes of Serum Melatonin, Interleukin-6, Homocysteine, and Complement C3 and C4 Levels in Patients with Depression. Front. Psychol. 11, 1271. 10.3389/fpsyg.2020.01271 32655450 PMC7324806

[B84] Tordjman S. Chokron S. Delorme R. Charrier A. Bellissant E. Jaafari N. (2017). Melatonin: Pharmacology, Functions and Therapeutic Benefits. Cn 15 (3), 434–443. 10.2174/1570159X14666161228122115 PMC5405617 28503116

[B85] Wain H. M. Bruford E. A. Lovering R. C. Lush M. J. Wright M. W. Povey S. (2002). Guidelines for Human Gene Nomenclature. Genomics 79 (4), 464–470. 10.1006/geno.2002.6748 11944974

[B86] Wasserman D. (2016). Review of Health and Risk-Behaviours, Mental Health Problems and Suicidal Behaviours in Young Europeans on the Basis of the Results from the EU-Funded Saving and Empowering Young Lives in Europe (SEYLE) Study. Psychiatr. Pol. 50 (6), 1093–1107. 10.12740/PP/66954 28211549

[B87] Wiechmann A. F. (2002). Regulation of Gene Expression by Melatonin: a Microarray Survey of the Rat Retina. J. Pineal Res. 33 (3), 178–185. 10.1034/j.1600-079x.2002.02935.x 12220334

[B13] World Health Organization. (2017). Depression and Other Common Mental Disorders: Global Health Estimates. https://www.who.int/mental_health/management/depression/prevalence_global_health_estimates/en/ (Accessed January 15, 2021).

[B92] Zaki N. F. W. Spence D. W. BaHammam A. S. Pandi-Perumal S. R. Cardinali D. P. Brown G. M. (2018). Chronobiological Theories of Mood Disorder. Eur. Arch. Psychiatry Clin. Neurosci. 268 (2), 107–118. 10.1007/s00406-017-0835-5 28894915

[B93] Zienolddiny S. Haugen A. Lie J.-A. S. Kjuus H. Anmarkrud K. H. Kjærheim K. (2013). Analysis of Polymorphisms in the Circadian-Related Genes and Breast Cancer Risk in Norwegian Nurses Working Night Shifts. Breast Cancer Res. 15 (4), R53. 10.1186/bcr3445 23822714 PMC3978690

